# Nucleotide Excision Repair Pathway Activity Is Inhibited by Airborne Particulate Matter (PM_10_) through XPA Deregulation in Lung Epithelial Cells

**DOI:** 10.3390/ijms23042224

**Published:** 2022-02-17

**Authors:** Ericka Marel Quezada-Maldonado, Yolanda I. Chirino, María Eugenia Gonsebatt, Rocío Morales-Bárcenas, Yesennia Sánchez-Pérez, Claudia M. García-Cuellar

**Affiliations:** 1 Subdirección de Investigación Básica, Instituto Nacional de Cancerología, San Fernando No. 22, Tlalpan 14080, Mexico; marelquezada0612@gmail.com (E.M.Q.-M.); mobarobiol@yahoo.com.mx (R.M.-B.); s_yesennia@yahoo.com.mx (Y.S.-P.); 2 Programa de Doctorado en Ciencias Biomédicas, Universidad Nacional Autónoma de México, Ciudad Universitaria, Coyoacán 04510, Mexico; 3 Unidad de Biomedicina, Facultad de Estudios Superiores Iztacala, Universidad Nacional Autónoma de México, Los Reyes Iztacala, Tlalnepantla de Baz 54090, Mexico; irasemachirino@gmail.com; 4 Departamento de Medicina Genómica y Toxicología Ambiental, Instituto de Investigaciones Biomédicas, Universidad Nacional Autónoma de México, Ciudad Universitaria, Coyoacán 04510, Mexico; mgonsebatt@gmail.com

**Keywords:** DNA adducts, DNA repair inhibition, nucleotide excision repair pathway, particulate matter, lung cancer

## Abstract

Airborne particulate matter with a diameter size of ≤10 µm (PM_10_) is a carcinogen that contains polycyclic aromatic hydrocarbons (PAH), which form PAH–DNA adducts. However, the way in which these adducts are managed by DNA repair pathways in cells exposed to PM_10_ has been partially described. We evaluated the effect of PM_10_ on nucleotide excision repair (NER) activity and on the levels of different proteins of this pathway that eliminate bulky DNA adducts. Our results showed that human lung epithelial cells (A549) exposed to 10 µg/cm^2^ of PM_10_ exhibited PAH–DNA adducts as well as an increase in RAD23 and XPD protein levels (first responders in NER). In addition, PM_10_ increased the levels of H4K20me2, a recruitment signal for XPA. However, we observed a decrease in total and phosphorylated XPA (Ser196) and an increase in phosphatase WIP1, aside from the absence of XPA–RPA complex, which participates in DNA-damage removal. Additionally, an NER activity assay demonstrated inhibition of the NER functionality in cells exposed to PM_10_, indicating that XPA alterations led to deficiencies in DNA repair. These results demonstrate that PM_10_ exposure induces an accumulation of DNA damage that is associated with NER inhibition, highlighting the role of PM_10_ as an important contributor to lung cancer.

## 1. Introduction

Outdoor air pollution, specifically particulate matter (PM), has an impact on the incidence and mortality of lung cancer worldwide [1,2]. Epidemiological evidence and research in animal and in vitro models supported the classification of PM as a class 1 carcinogen by the International Agency for Research on Cancer (IARC) [3,4]. PM is divided according to its aerodynamic diameters into PM_10_ (≤10 µm), PM_2.5_ (≤2.5 µm), and ultrafine particles (UFPs) (≤0.1 μm). PM_10_ contains all of the smaller fractions, and all of them are deposited in the respiratory tract during breathing [5,6,7,8,9]. PM_10_ is made up of a variety of metals, such as zinc, copper, and vanadium; biological agents, such as pollen, bacteria, and endotoxins; and polycyclic aromatic hydrocarbons (PAH), including benzo(g)perylene (BghiP), dibenzo(a)anthracene (DBahA), and benzo(a)pyrene (BaP). The composition of PM_10_ plays an important role in its genotoxic and carcinogenic potential because some of these components modify the DNA [10,11].

There are several studies in which the role of PM_10_ components as mediators in the DNA damage has been demonstrated. For instance, metals can mediate an increase in reactive oxygen species (ROS), which, in turn, leads to the oxidation of DNA [12,13]. In this regard, base excision repair (BER) is the pathway responsible for repairing the oxidative DNA lesions that cause small distortions in the helical structure of DNA, but enzymatic and non-enzymatic antioxidant defense also protects cells from the ROS generation induced by PM_10_ exposure [14,15]. However, some other components of PM_10_, such as PAH, mediate genotoxic effects through the induction of bulky DNA lesions, mainly BaP, which is biotransformed by cytochrome P450 enzymes in combination with microsomal epoxide hydrolase, leading to the generation of the reactive species benzo(a)pyrene-7,8-diol-9,10-epoxide (BPDE), which can form adducts with DNA (BPDE–DNA) and cause significant distortions in the helical structure of this biomolecule [16,17,18,19,20,21]. The antioxidant defenses and the BER pathway are insufficient for coping with such distortions. The nucleotide excision repair (NER) pathway, which is composed of more than 30 proteins, acts in the removal of bulky lesions formed in the DNA, including the PAH–DNA adducts, through one of two sub-pathways: the global-genome (GG-NER) pathway or the transcription-coupled (TC-NER) pathway [22,23].

Damage recognition is the first step of the pathway and is carried out by the XPC-RAD23 proteins in GG-NER. Unwinding of the DNA strand is the next step and is performed by the helicases XPD and XPB [22,24]. A DNA damage verification step is carried out by the XPA protein, which is a key scaffold protein required for the verification of lesions and for the recruitment of other NER pathway proteins. Proper binding of XPA to the site of DNA damage depends on histone 4 (H4), which is dimethylated in lysine 20 (H4K20me2) in the presence of bulky DNA lesions [25,26,27]. Together with RPA, XPA forms the pre-incision complex in damaged DNA, which ensures that lesions are properly excised and thus represents an important rate-limiting step. In the third step, the XPF-ERCC1 complex cleaves the damage at the 5′-DNA strand, whereas the 3’ incision is mediated by the XPG endonucleases; at the end, δ or ε DNA polymerases synthesize the new DNA strand using the undamaged strand as a template [28].

Post-translational modifications regulate DNA repair by modulating some factors of the NER pathway, including XPA [29]. The phosphorylation of XPA at serine residue 196 (pXPA^S196^) by kinase ATR enhances the stability of XPA and is required for the formation of the XPA-RPA protein complex. WIP1 phosphatase catalyzes the dephosphorylation of XPA at S196, thus inactivating this protein and reducing the NER pathway’s functionality, which might increase the carcinogenic potential of different compounds that cause DNA damage [30,31,32,33,34,35]. DNA adducts can modify DNA conformation and deregulate replication and transcription. Therefore, the accumulation of this type of DNA lesion can induce genomic instability, lead to the appearance of mutations, and promote carcinogenic processes [36,37,38]. In relation to this, alterations in the NER repair system have been described in different types of cancer, including lung neoplasms [39,40]. However, the effect of PM_10_ exposure in the NER pathway remains unclear. The aim of this study was to investigate the deregulation of the RAD23, XPD, XPA, pXPA^s196^, H4k20me2, and WIP1 proteins, which are the main components of NER pathway responsible for the removal of bulky DNA damage, establishing an association with the NER pathway activity in A549 lung epithelial cells exposed to PM_10_ for 24 and 48 h.

## 2. Results

### 2.1. PM_10_ Induced the Formation of BPDE–DNA Adducts

**Figure 1:**
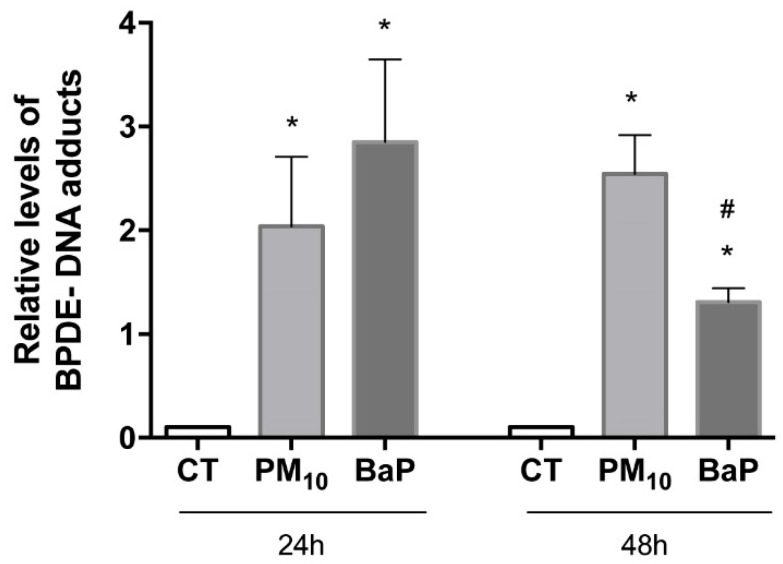
PM_10_ exposure induces the formation of BPDE–DNA adducts in A549 cells. Benzo(a)pyrene diol epoxide-DNA (BPDE–DNA) adducts were evaluated in A549 cells exposed to 10 µg/cm^2^ of PM_10_ and 1 µM of BaP for 24 and 48 h. The concentration of BPDE–DNA adducts was measured using the OxiSelect BPDE DNA Adduct ELISA Kit and expressed according to the relative levels, and the values represent results from three experiments with the mean ratio ± SD per treatment. BaP was used as a positive control for DNA adduct generation. The images are representative of the data obtained. (*) indicates statistical differences versus the control group; *p* < 0.05. (#) indicates statistical differences between the amounts of time; *p* < 0.05.

PM_10_ exposure for 24 h increased the levels of BPDE–DNA adducts in A549 cells compared with the control group (FC = 2.01 vs. 0.0; *p* < 0.05) (Figure 1). PM_10_ exposure for 48 h showed a further increase in BPDE–DNA adducts (FC = 2.54 vs. 0.0; *p* < 0.05). BaP exposure for 24 h increased the levels of BPDE–DNA adducts in A549 cells compared with the control group (FC = 2.85 vs. 0.0; *p* < 0.05). BaP exposure for 48 h also showed an increase in BPDE–DNA adducts (FC = 1.30 vs. 0.0; *p* < 0.05). The comparison of the times exhibited a significant decrease in the levels of BPDE–DNA adducts among cells treated with BaP for 48 h compared with that in those treated for 24 h (FC = 1.30 vs. 2.85; *p* < 0.05).

### 2.2. PM_10_ Deregulated the RAD23, XPD, and XPA Proteins Used in the Recognition and Verification Step of the NER Pathway

**Figure 2:**
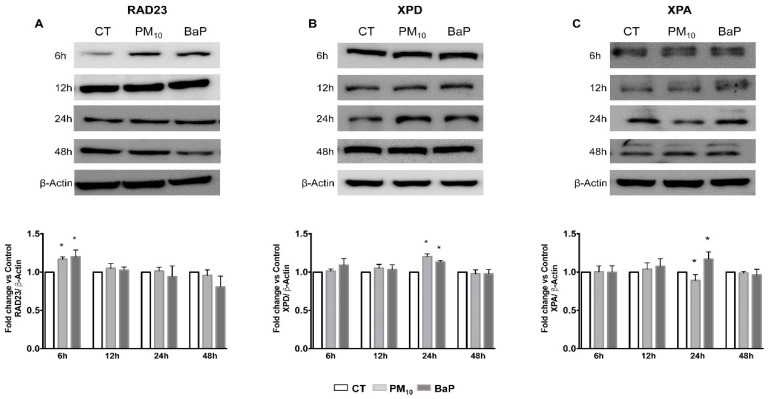
PM_10_ deregulated proteins used in the NER pathway during different stages of exposure in A549 cells. The protein levels of (**A**) RAD23, (**B**) XPD, and (**C**) XPA were evaluated with a Western blot in the total protein lysates of A549 lung epithelial cells exposed to 10 µg/cm^2^ of PM_10_ and 1 µM of BaP for 6, 12, 24, and 48 h. Representative images of protein levels in protein lysates (upper panels) and an analysis of densitometry levels (lower panels) using β-Actin as a housekeeping control are shown. β-Actin blot housekeeping control is representative of all time point experiments (see Supplementary Figure S1). The values represent results from three independent experiments with the mean ± SD per treatment. BaP was used as a positive control for NER pathway activation. The images are representative of the data obtained. (*) indicates statistical differences versus the control group; *p* < 0.05.

PM_10_ exposure for 6 h increased the RAD23 protein levels in A549 cells compared with the control group (1.16 vs. 1.00; *p* < 0.05) (Figure 2A). BaP exposure for 6 h also increased the RAD23 protein levels compared with the control group (1.20 vs. 1.00; *p* < 0.05). No differences were observed in the RAD23 protein levels after PM_10_ or BaP exposure for 12 h (1.04 and 1.02 vs. 1.00, respectively), 24 h (1.01 and 0.94 vs. 1.00, respectively), or 48 h (0.95 and 0.90 vs. 1.00, respectively) compared with their control groups.

No differences were observed in the XPD protein level after PM_10_ or BaP exposure for 6 h (1.01 and 1.09 vs. 1.00, respectively), 12 h (1.05 and 1.03 vs. 1.00, respectively), or 48 h (0.97 and 0.98 vs. 1.00, respectively) compared with the control groups (Figure 2B). However, PM_10_ exposure for 24 h increased the XPD protein level in A549 cells compared with the control group (1.20 vs. 1.00; *p* < 0.05). BaP exposure for 24 h also increased the XPD protein level compared with the control group (1.15 vs. 1.00; *p* < 0.05).

No differences were observed in the XPA protein level after PM_10_ or BaP exposure for 6 h (1.00 and 0.99 vs. 1.00, respectively), 12 h (1.03 and 1.07 vs. 1.00, respectively), or 48 h (0.98 and 0.96 vs. 1.00, respectively) compared with the control groups (Figure 2C). However, PM_10_ exposure for 24 h decreased the XPA protein level in A549 cells compared with the control group (0.85 vs. 1.00; *p* < 0.05). On the other hand, BaP exposure for 24 h increased the XPA protein level compared with the control group (1.17 vs. 1.00; *p* < 0.05).

### 2.3. PM_10_ Induced Nuclear Recruitment (H4K20me2) and Dephosphorylation of XPA Associated with WIP1 Increase

**Figure 3:**
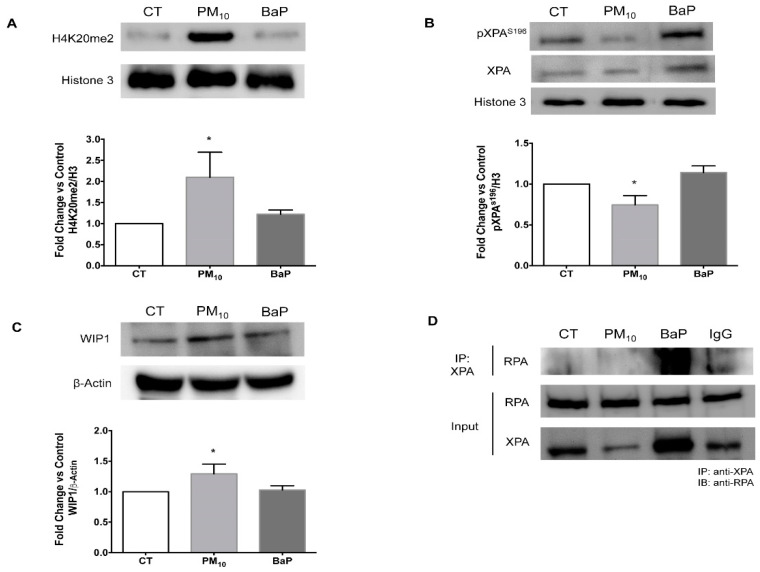
PM_10_ exposure induced signals for the recruitment of XPA into the nucleus but decreased its phosphorylation, thus inhibiting the formation of the XPA-RPA complex in A549 cells. Protein levels were evaluated with a Western blot in nuclear protein lysates of A549 lung epithelial cells after exposure to 10 µg/cm^2^ of PM_10_ and 1 µM of BaP for 24 h. BaP was used as a positive control for NER pathway activation. (**A**) Representative Western blot of H4K20me2 in nuclear protein lysates (upper panel) and levels of a densitometry analysis using H3 as an endogenous control (bottom panel). (**B**) Representative Western blot of pXPA^S196^ in cytoplasm and nuclear protein lysates, total XPA is displayed (upper panel) and levels of a densitometry analysis using histone 3 as an endogenous control (bottom panel). (**C**) Representative Western blot of WIP1 levels in total protein lysates (upper panels) and levels of a densitometry analysis using β-Actin as an endogenous control (bottom panels). The images and values represent results from three independent experiments with the mean ± SD per treatment. The images are representative of the data obtained. (*) indicates statistical differences versus the control group; *p* < 0.05. (**D**) Representative Western blot of the interaction between XPA and RPA detected by an immunoprecipitation assay after 24 h. IP: immunoprecipitation, IB: immunoblot. Representative image of three independent experiments.

Cells treated with PM_10_ for 24 h exhibited an increase in nuclear H4K20me2 protein levels compared with the control group (2.09 vs. 1.00; *p* < 0.05), whereas cells treated with BaP for 24 h showed no statistically significant differences (1.10 vs. 1.00) (Figure 3A). In addition, cells treated with PM_10_ for 24 h exhibited a decrease in nuclear pXPA^S196^ protein levels compared with the control group (0.74 vs. 1.00; *p* < 0.05), whereas cells treated with BaP for 24 h showed no differences (1.13 vs. 1.00) (Figure 3B). Interestingly, cells treated with PM_10_ for 24 h exhibited an increase in phosphatase WIP1 protein levels compared with the control group (1.29 vs. 1.00; *p* < 0.05), whereas cells treated with BaP for 24 h showed no differences (1.02 vs. 1.00) (Figure 3C).

### 2.4. PM_10_ Impaired the Formation of the XPA-RPA Complex

Cells treated with PM_10_ for 24 h did not display the XPA-RPA interaction, indicating that the complex between these proteins is not formed, whereas cells treated with BaP for 24 h showed an effective interaction between XPA and RPA, exhibiting a successful formation of this complex (Figure 3D).

### 2.5. The NER Pathway Was Inactive in Cells Exposed to PM_10_

**Figure 4:**
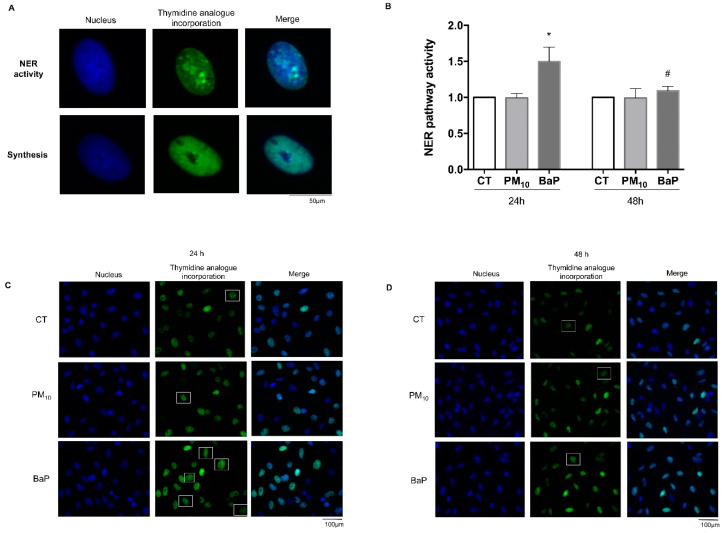
The NER pathway is inactive in A549 cells that are exposed to PM_10._ The NER pathway’s activity was evaluated through unscheduled DNA synthesis (UDS) in A549 lung epithelial cells exposed to 10 µg/cm^2^ of PM_10_ and 1 µM of BaP for 24 and 48 h. (**A**) The panel shows representative fluorescence micrographs of the detection of the incorporation of thymidine analog (EdU), and NER pathway activity is recognized according to the formation of foci (upper panel). Non-specific incorporation during DNA synthesis was identified by green homogeneous nucleus staining (lower panel), and these cells were not considered in the cell count for the repair analysis. (**B**) The quantitative results of the NER pathway activity were expressed after counting 1500 cells per condition. The values represent results from three experiments with the mean ratio ± SD per treatment. BaP was used as a positive control for NER pathway activation. (*) indicates statistical differences versus the control group; *p* < 0.05. (#) indicates statistical differences between the amounts of time compared; *p* < 0.05. (**C**,**D**) Representative images of NER pathway activity (positive cells are marked in white squares) at 24 and 48 h, respectively. The magnification of the NER pathway activity panels can be observed in Supplementary Figure S2 (24 h) and Supplementary Figure S3 (48 h).

Cells treated with PM_10_ for 24 and 48 h showed no differences in NER pathway activity compared with the control groups (0.99 vs. 1.00, and 0.99 vs. 1.00, respectively) (Figure 4). Cells treated with BaP for 24 h showed an increase in NER pathway activity compared with the control group (1.50 vs. 1.00; *p* < 0.05), whereas cells treated with BaP for 48 h showed no differences in NER pathway activity compared with the control group (1.09 vs. 1.00). The comparison between the amounts of time exhibited a significant decrease in NER pathway activity among cells treated with BaP for 48 h compared with those treated for 24 h (1.09 vs. 1.50; *p* < 0.05).

## 3. Discussion

PM_10_ is a well-known risk factor for the development of lung cancer [5,7]. Some of the components of PM_10_ contained in this complex mixture are highly toxic or have been classified as carcinogens, including metals and PAH [17]; however, until now, a mutational fingerprint associated with PM exposure has not been detected [41]. Therefore, the study of the genotoxicity and effects of PM on DNA damage repair pathways could help in understanding the mechanism of PM_10_ in lung carcinogenesis because the evasion of DNA repair induces the accumulation of damaged DNA. It is mainly the altered activity of NER, which is responsible for repairing bulky lesions, that leads to cancer [19,24,42,43]. In this study, A549 cells were exposed to a sub-lethal concentration of 10 µg/cm^2^, which simulated human PM_10_ exposure for five days [44,45], and the effects on proteins and the functionality of NER were analyzed.

We showed that PM_10_ induced the formation of BPDE-DNA, and the literature indicated that 60% of bulky lesions are removed within 48 h of their generation [46,47,48]; however, in cells exposed to PM_10,_ these lesions persisted for over 48 h. By contrast, when the same cell line was exposed to BaP, it showed a decrease in BPDE–DNA adducts at 48 h, suggesting that more than half of the BPDE adducts were eliminated. This highlights that DNA damage induced by PM_10_ could be accumulated more in comparison with the DNA damage induced by BaP, despite being a well-characterized carcinogen. The second main finding of our study is related to a detailed identification of the proteins in the NER pathway. When each step of the repair was evaluated by determining the levels of key NER pathway proteins, we found that in cells exposed to PM_10_, the RAD23, H4K20me2, and XPD proteins increased, whereas there was a decrease in XPA protein.

NER activity studies have shown that in A549 cells exposed to PAH, the beginning of DNA repair occurs 4 to 6 h after the generation of DNA damage [49]. Therefore, the increase in the RAD23 protein that we observed in cells exposed to PM_10_ in the first hours after exposure suggested that the cells could recognize DNA damage. In addition, in this work, no changes were observed in RAD23 in the following hours of exposure in either PM_10_ or BaP, which indicates that this protein is only necessary during the initial repair step, as has been demonstrated in other studies [26,28,50]. It was also found that after damage recognition, the DNA probably unwinds in cells exposed to PM_10_ because of the increase in the XPD protein, which functions as helicase [27]. Furthermore, PM_10_ increased the levels of H4K20me2, confirming that in cells exposed to PM_10_, the damaged DNA recognition step works correctly, so this could induce the recruitment of other proteins, such as XPA [25]. In cells exposed to BaP, an increase in XPD levels was found; however, no changes were detected in H4K20me2 levels, so it is likely that this event occurred hours before the protein was measured; further experiments need to be conducted to determine these changes.

Alterations in the XPA protein generate a disruption in the progression of the NER pathway because although XPA does not possess enzymatic activity, it plays a critical role in the assembly of the pre-incision complex in damaged DNA [51]. Importantly, the levels of XPA decrease in cells exposed to PM_10_ even though the recruitment signal for XPA is active, as suggested by the increase in H4K20me2 levels. However, in cells exposed to BaP, an increase in XPA levels was observed, suggesting that in cells exposed to PM_10_, the verification step is altered. We proposed that the decrease in XPA in cells exposed to PM_10_ could be a consequence of the metals contained in PM_10_ because XPA is structured by zinc fingers [52], and nickel, zinc, cadmium, and copper can oxidize the thiol groups of these domains, thus inducing conformational and structural changes in this protein [48,52,53,54]. In addition, the reactive oxygen species produced by PM_10_ exposure could also be responsible for alterations in enzymatic activity [55]. We must emphasize that PM_10_ exposure decreased the rate of phosphorylation of XPA, suggesting that PM_10_ alters the function of the XPA protein because dephosphorylated XPA loses its ability to interact with other proteins [33,56]. In addition, altered phosphorylation can lead to a decrease in XPA level, because dephosphorylated XPA is a substrate for HERC2 ubiquitination and proteasome degradation [30,33].

The loss of phosphorylation of XPA in serine 196 observed in this study could be the result of the increase in PM_10_-induced WIP1 phosphatase levels; Nguyen et al. reported that WIP1 dephosphorylates the serine/threonine residues of different repair proteins, including XPA [31]. In addition, the cells exposed to BaP did not show alterations in either the levels of pXPA^S196^ or WIP1. Cells expressing higher levels of WIP1 have shown reduced repair kinetics for the NER pathway [31,57], and WIP1 overexpression has been reported in some tumors, including lung adenocarcinoma [58,59]. We suggest that PM_10_ exposure could stimulate the phosphatase activity of WIP1 because the functionality of phosphatases depends on low concentrations of metals, such as magnesium or manganese [60,61], and PM_10_ has been shown to contain these metals [18,20]. We propose that the dephosphorylation of XPA induced by exposure to PM_10_ has an impact on the interaction between XPA and RPA because this complex was absent in cells exposed to PM_10_. Since this complex performs the verification of damage and recruitment of the excision proteins, such as ERCC1 [51,62,63], we suspect that in cells exposed to PM_10_, the DNA adducts could not be adequately removed during this step of the NER pathway, in contrast to cells exposed to BaP, which showed a higher rate of DNA adduct removal associated with the presence of the XPA-RPA complex.

Because we observed the persistence of DNA adducts in cells exposed to PM_10_ and we also found alterations in XPA, one of the main proteins of the NER pathway, we decided to measure the activity of the NER pathway. Through this analysis, we confirmed that despite the recognition of damage, the functioning of the NER pathway was inhibited after exposure to PM_10_. On the contrary, cells exposed to BaP showed a clear increase in the activity of the NER pathway, which is consistent with the decrease in the concentration of BPDE adducts found at 48 h. Therefore, the use of BaP as a positive damage control allowed us to determine that the NER pathway works correctly in A549 cells. The inadequate ability to remove damage indicates that alterations in XPA levels and in the phosphorylation reduce the response of the NER pathway in A549 cells exposed to PM_10_, highlighting the likely role for WIP1 in inhibition of DNA repair; these are findings that need to be confirmed later. BaP, a carcinogenic component of PM_10_, had no effect on NER pathway activity, which highlights that the effects on DNA repair activity might be the result of synergistic effects of all PM_10_ components [20].

On the other hand, it is highly likely that NER is not the only DNA repair pathway disrupted by PM_10_ exposure. There is clear evidence of PM_10_-induced DNA damage, including the detection of 8-hydroxy-2-deoxyguanosine, DNA strand breaks, and formation of γH2AX foci [12,64,65], which is also supported by indirect evidence of DNA damage, such as micronucleus formation [66]; however, the accuracy of the alterations in proteins involved in the DNA repair pathways is still being assessed. For now, some hints of protein dysregulation of the BER pathway, homologous recombination (HR), and nonhomologous end-joining (NHEJ) pathways suggest potential impairment in the global DNA repair after PM_10_ exposure [67]. Together, all DNA repair pathways protect the genome and its fidelity, but according to the literature, deficiencies in the NER and mismatch repair pathways have greater implications for carcinogenesis, whereas alterations in the BER pathway have a very low impact on cancer development [68,69].

**Figure 5:**
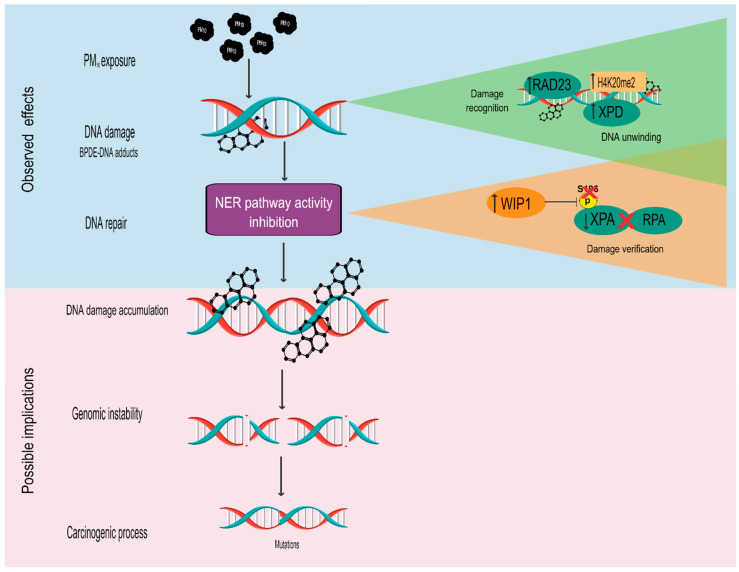
Schematic representation of the possible effects of PM_10_ exposure associated with NER pathway inactivation. Exposure to PM_10_ induces DNA damage through the formation of DNA adducts and this damage is recognized by the overexpression of RAD23, which induces the activity of the XPD and H4K2me2 proteins. Nevertheless, the removal of these adducts is inhibited as a result of the decrease in XPA levels and their dephosphorylation at serine residue 196 mediated by the upregulation of WIP1, which, in turn, disrupts the formation of the complex between XPA and RPA. Therefore, we suggest that the alteration in the functioning of the NER pathway predisposes cells to the accumulation of DNA damage and contributes to genomic instability, which could lead to the generation of mutations and, ultimately, to carcinogenic processes mediated by PM_10_, which could be aggravated if some other DNA repair pathways are also inactivated.

Therefore, although additional studies are needed to assess the significance of the results of our study, the data indicate that lung cells exposed to PM_10_ can accumulate DNA damage, which could predispose cells to genomic instability, and, in turn, this could lead to carcinogenesis [70,71]. PM_10_ exposure induces the formation of DNA adducts, and their removal by the NER pathway is impaired in lung epithelial cells so that the risk of lung cancer development attributed to PM_10_ inhalation can be explained by DNA accumulation more than by mutations in early stages of exposure. However, we do not discard that DNA accumulation induced by PM_10_ exposure could lead to mutations that are still not detected (Figure 5).

## 4. Materials and Methods

### 4.1. PM_10_ Collection

PM_10_ was collected from a residential urban area of Mexico City, one of the main sources of air pollution in the city, using a high-volume air collector (GMW model 1200 VFC HVPM10 Sierra Andersen, Smyrna, GA, USA) with a constant flow of 1.13 m^3^/min. To recover PM_10_, we used nitrocellulose filters with a pore size of 3.0 μm (Sartorius AG, Goettingen, Germany), which were then scraped with a surgical blade to collect the PM. PM_10_ was stored in endotoxin-free glass vials at 4 °C in the dark until use. The PM_10_ utilized in this study was characterized in previous studies through the analysis of PAH, metals, and endotoxins [72].

### 4.2. Cell Culture and PM_10_ Exposure

The A549 human lung epithelial cell line was purchased from the American Type Culture Collection (ATCC, Manassas, VA, USA) and was cultured in F-12 Kaighn’s medium (Gibco BRL, 21127022, Grand Island, NY, USA) supplemented with 10% heat-inactivated fetal bovine serum (FBS; GIBCO, 16000044, Life Technologies, Brooklyn, NY, USA) at 37 °C using a 5% CO_2_ atmosphere. One milligram of the stock suspension of PM_10_ was resuspended in one milliliter of F-12 Kaighn’s medium to obtain a PM_10_ suspension of 1 mg/mL, as previously described [73]. After reaching 70% confluence, cells were exposed to 10 μg/cm^2^ of PM_10_ in F-12K medium supplemented with 10% FBS. Cells with only F-12K medium supplemented with 10% FBS were used as a control (CT), and cells treated with BaP (1 μM) (Sigma, B1760, USA) were used as a positive control for DNA adduct generation and the activation of the NER pathway [19,74].

### 4.3. Measurement of the Benzo(a)pyrene-7,8-diol-9,10-epoxide-DNA Adducts (BPDE-DNA Adduct)

After the cells were exposed to PM_10_ and BaP for 24 or 48 h, DNA was isolated using the phenol–chloroform–isoamyl alcohol extraction protocol of Sambrook et al., 1989 [75]. DNA was dissolved in nuclease-free water and quantified using an ND-1000 spectrophotometer (NanoDrop Technologies, Wilmington, NC, USA). The DNA integrity was evaluated using agarose gel electrophoresis. The BPDE-DNA concentration was measured using the OxiSelect BPDE DNA Adduct ELISA Kit (Cell Bio-labs, Inc., STA-357, San Diego, CA, USA) using the BPDE-DNA standard curve, according to the manufacturer’s protocol. The absorbance was read in fluorescence plate reader (Tecan, GENios Plus, Männedorf, Switzerland) at 450 nm. The results were expressed as relative levels represented as fold changes (FCs) based on the calculation of nanograms of BPDE–DNA adducts per microgram of DNA.

### 4.4. Evaluation of the Total Protein Levels of the NER Pathway

The protein levels of RAD23, XPD, XPA, and WIP1 were evaluated at 6, 12, 24, and 48 h in cells exposed to PM_10_ and BaP. Cells were washed with PBS, and protein extraction was performed using RIPA lysis buffer (20 mM Tris pH 8.0, 1% NP-40, and 150 mM NaCl at pH 8.0) with protease and phosphatase inhibitors (Thermo Fisher, 78440, Rockford, IL, USA). Protein quantification was performed by using the bicinchoninic acid method with a bovine serum albumin curve as a standard (Thermo Fisher, 23209, California, UK). Thirty micrograms of protein were used for electrophoresis on 12% SDS polyacrylamide gels, and the proteins were transferred to 0.45 µm polyvinylidene difluoride (PVDF) membranes using a semidry blotting system (Trans-Blot-Turbo, transfer system; Bio-Rad, California, UK). Membranes were blocked with 5% low-fat milk in TBS-Tween 0.1% under agitation for 1 h. Primary antibodies were incubated in a dilution of 1:1000 (anti-RAD23 cell signaling, 24555, anti-XPD cell signaling, 11963, anti-XPA Santa Cruz, sc-56497, and anti-WIP1 cell signaling, 11901) overnight at 4 °C under constant agitation. Anti-beta-actin (β-Actin) was used as a housekeeping protein in a dilution of 1:3000 (monoclonal antibody donated by Dr. Manuel Hernández, Cinvestav-IPN) [64,76]. After incubation, membranes were washed with TBS–Tween 0.1% and incubated with HRP-secondary anti-rabbit antibody (Amersham, NA934V) 1:2000 or HRP-secondary anti-mouse antibody (Amersham, NA931) 1:3000 for 1 h. Immunodetection was performed with chemiluminescence peroxidase substrate (Millipore, WBKLS0100, Billerica, MA, USA) and with the ChemiDoc-It Imager UVP. A densitometry analysis was performed by using the Image J software.

### 4.5. Measurements of Nuclear Protein Levels of the NER Pathway

The nuclear protein levels of H4K20me2 and XPA Ser196 were evaluated after the A549 cells were exposed to PM_10_ and BaP for 24 h. Protein extraction was performed by separating the nuclear protein fraction and cytoplasmic protein fraction using Chemicon’s nuclear extraction kit (Millipore, 2900, Billerica, MA, USA) according to the manufacturer’s instructions. Protein quantification was performed using the bicinchoninic acid assay, as previously mentioned. Fifteen micrograms of nuclear protein fraction was loaded into a 15% SDS-polyacrylamide gel, and the levels of proteins were determined as previously described. Anti-H4K20me2 antibody (Abcam, ab9052) at 1:2000 and anti-phospho-XPA (Ser196) antibody (Thermo Fisher, 64730) at 1:500 were incubated overnight at 4 °C under constant agitation. Histone 3 (H3) (Abcam, ab1791) was used as a housekeeping protein at 1:5000 for 1 h at room temperature, followed by the incubation of HRP-secondary anti-mouse antibody (Amersham, NA931).

### 4.6. Detection of the XPA-RPA Protein Complex

The immunoprecipitation assay was performed using the Dynabeads Co-Immunoprecipitation kit (Invitrogen, Thermo Fisher Scientific, 14321D) according to the manufacturer’s protocol for detection of the XPA-RPA complex. One milligram of magnetic beads and 5 µg of XPA antibody (Santa Cruz, sc-56497) or 5 µg of normal goat IgG antibody (R&D systems, AB-108-C) were used for the formation of complex ab-magnetic beads. One milligram of protein cell lysates was incubated with the complex ab-magnetic beads. The elution of the immune complex was electrophoresed on 12% SDS-polyacrylamide gel, according to the steps described in the protein determination subsection. Anti-RPA (cell signaling, 2267) antibody at 1:1000 and HRP-secondary anti-rabbit antibody (Amersham, NA934V) at 1:3000 were used.

### 4.7. Measurement of NER Activity

The activity of the NER pathway was evaluated with an unscheduled DNA synthesis (UDS) assay. This technique provides a direct measurement of the excision and repair of damage after in vitro exposure to the compounds of interest by incorporating the thymidine analogue (5-ethinyl-2’-deoxyuridine (EdU) into DNA during the synthesis of the new strand in the final step of NER [77,78]. DNA replication was blocked (for prevention of nonspecific incorporation of EdU) by incubating the cells with 5 mM hydroxyurea for 2 h before the treatments, and it was left in the culture medium until the end of the corresponding exposure [79]. Subsequently, the cells were exposed to PM_10_ or BaP, and 10 μM EdU was added to the culture medium. After 24 and 48 h of exposure, cells were washed with PBS and fixed using 3.7% paraformaldehyde in PBS for 20 min at room temperature. The incorporation of EdU was visualized with fluorescence microscopy using the Click-iT EdU Imaging Kit (Invitrogen, C10337, Carlsbad, CA, USA) according to the manufacturer’s recommendations. At the end of the procedure, the slides were dried, and the nuclei were stained with Prolong Gold antifade DAPI (Invitrogen, 8961S, CA, USA). The UDS slides were observed under an AxioKop2 Mot Plus.D2 fluorescence microscope (Carl Zeiss, Oberkochen, Germany). Micrographs of the different treatments were taken, and the number of EdU-positive cells (foci present in the nucleus) was counted among 1500 cells. Cells that presented a complete staining of their nuclei with EdU (incorporation during the replication of DNA) were excluded from the analysis [78,80] (Figure 4). The activity of the NER pathway was calculated as the percentage of EdU-positive cells in each treatment divided by the percentage of EdU-positive cells present in the control cells.

### 4.8. Statistical Analysis

The results of at least three independent experiments are presented as means ± standard deviation (SD). Statistical differences between DNA adducts and NER activity were tested by using one way analysis of variance and Bonferroni’s post hoc test. Protein levels were tested by applying the two-tailed Student’s t-test. All analyses were performed using the GraphPad Software, version 6 and a value of *p* ≤ 0.05 was considered statistically significant.

## 5. Conclusions

Exposure to PM_10_ induces the formation of BPDE–DNA adducts, which are recognizable by RAD23; however, PM_10_ deregulates the damage verification step through the dephosphorylation of XPA at serine 196, thus preventing the formation of the protein complex with RPA, which results in the inhibition of the NER pathway’s activity in A549 cells. These findings provide evidence that the impairment of the NER pathway’s activity and the damaged DNA might be involved in the carcinogenic potential of airborne particulate matter, thus helping explain why PM_10_ is considered a risk factor for the development of lung cancer.

## Data Availability

The data that support the findings of this study are available from the corresponding author upon reasonable request.

## Supplementary Materials

The following supporting information can be downloaded at: https://www.mdpi.com/article/10.3390/ijms23042224/s1.
