# Supplementary material for: Nucleotide Excision Repair Pathway Activity Is Inhibited by Airborne Particulate Matter (PM10) through XPA Deregulation in Lung Epithelial Cells
Source: Int J Mol Sci. 2022 Feb 17;23(4):2224. doi: 10.3390/ijms23042224 (PMC8878008; doi:10.3390/ijms23042224)
Supplement: Supplementary file 1 [file ijms-23-02224-s001.zip › ijms-1582382-Supplementary.pdf]

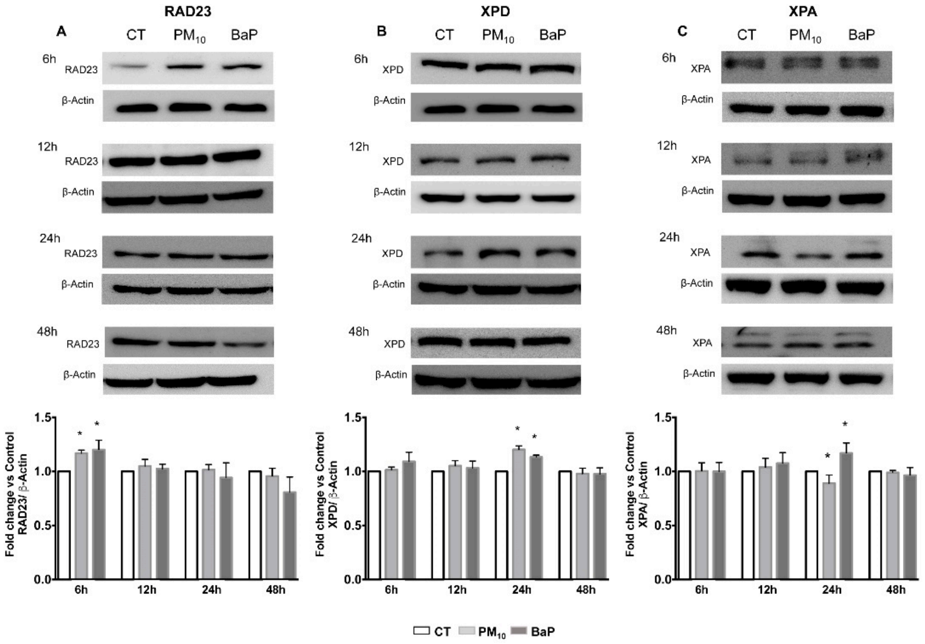

Supplementary Figure S1. PM_{10} deregulated proteins used in the NER pathway during different stages of exposure in A549 cells. The protein levels of (A) RAD23, (B) XPD, and (C) XPA were evaluated with a Western blot in the total protein lysates of A549 lung epithelial cells exposed to 10 μg/cm^2 of PM_{10} and 1 μM of BaP for 6, 12, 24, and 48 h. Representative images of protein levels in protein lysates and their respective β-Actin using as housekeeping control (upper panels) and the analysis of densitometry levels (lower panels). The values represent results from three independent experiments with the mean ± SD per treatment. BaP was used as a positive control. The images are representative of the data obtained. (*) indicates statistical differences versus the control group; $p < 0.05$

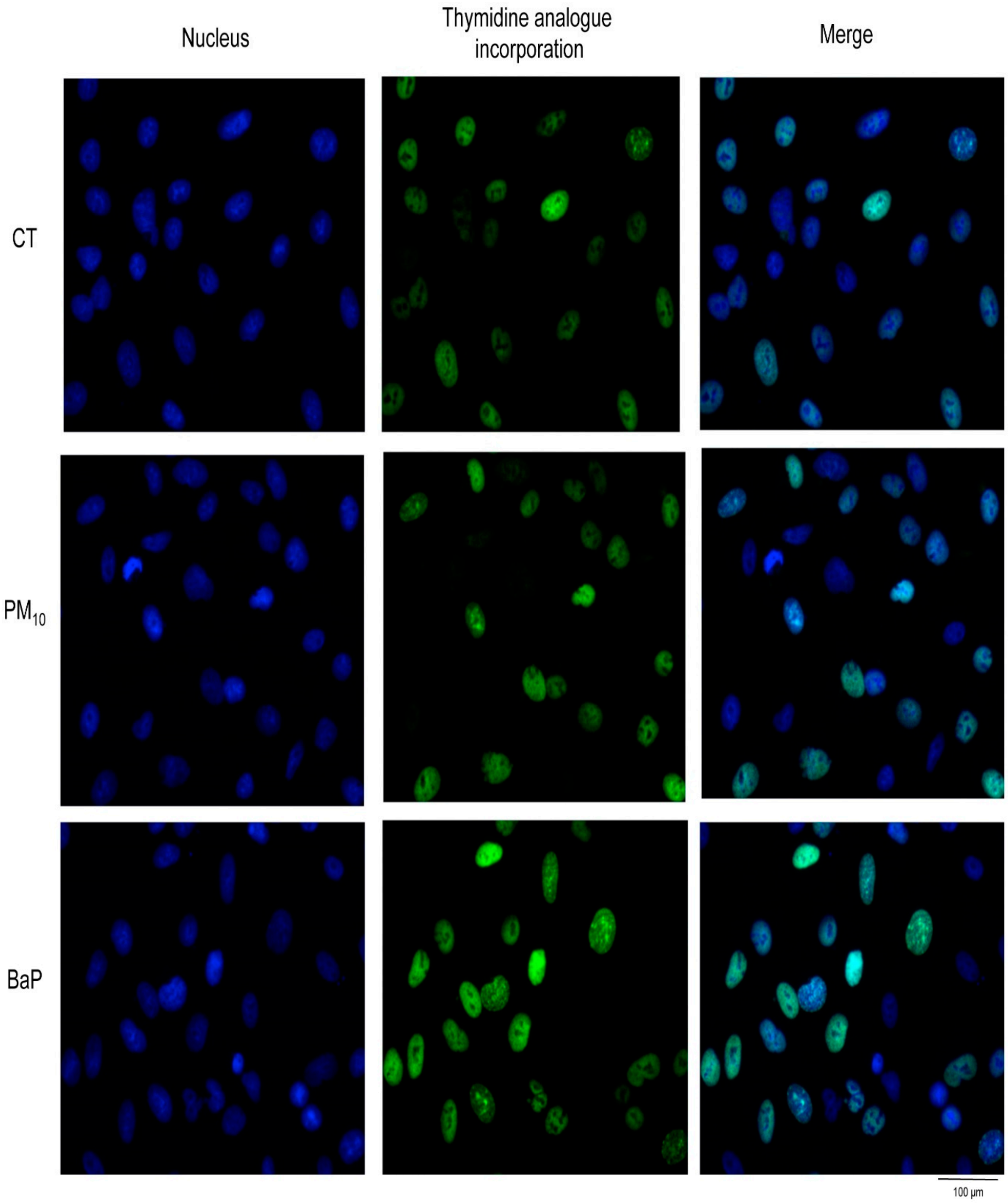

Supplementary Figure S2. The NER pathway is inactive in A549 cells that are exposed to PM_{10} for 24 h. The NER pathway´s activity was evaluated through unscheduled DNA synthesis (UDS) in A549 lung epithelial cells exposed to 10 µg/cm² of PM_{10} and 1 µM of BaP. Amplified panels of representative images of NER pathway activity after 24 h of exposure, recognized in cell that incorporated thymidine analogue (EdU) forming repair foci. Non-specific incorporation during DNA synthesis was identified by green homogeneous nucleus staining and these cells were not considered in the cell count for the repair analysis.

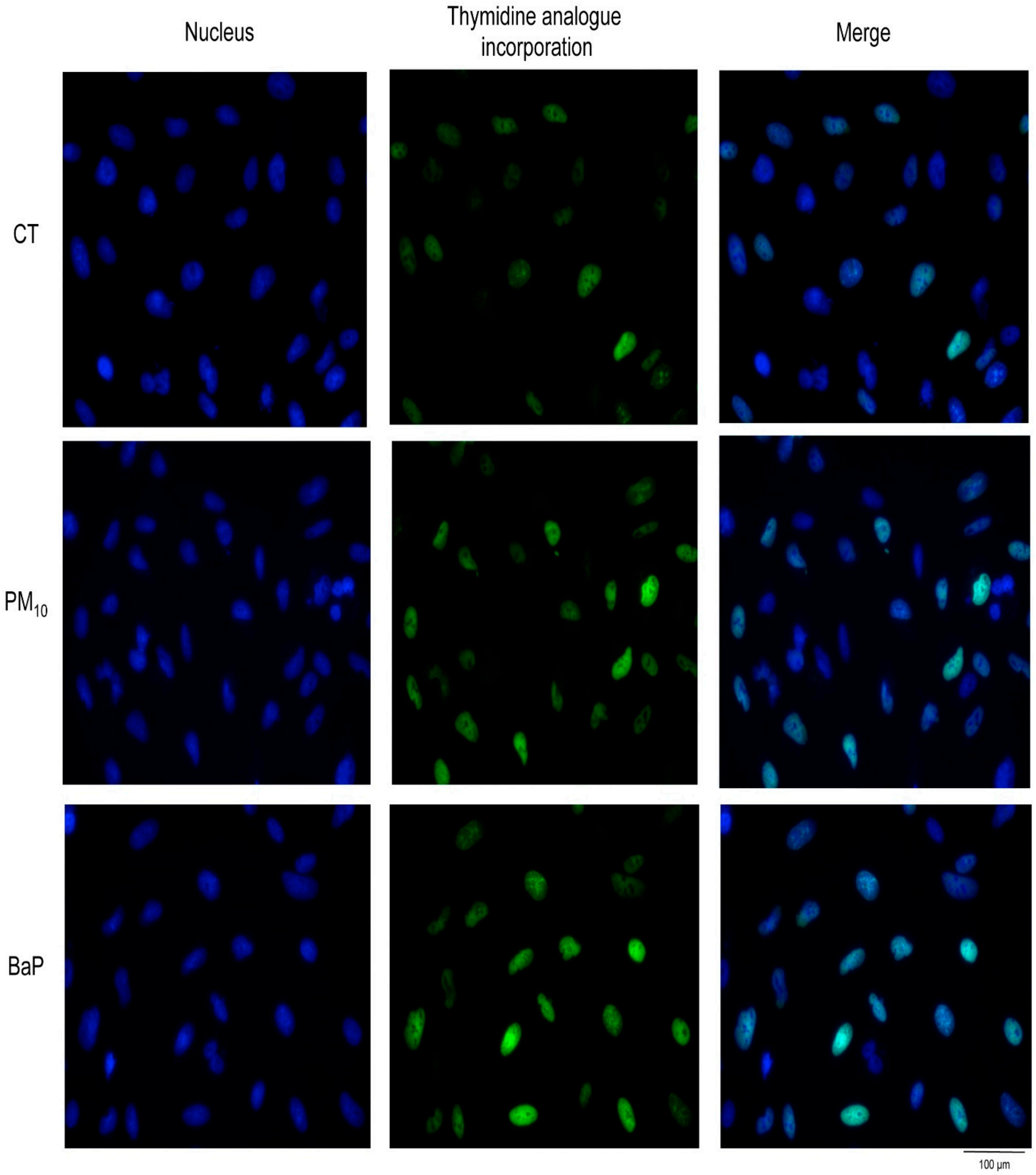

Supplementary Figure S3. The NER pathway is inactive in A549 cells that are exposed to PM_{10} for 48 h. The NER pathway´s activity was evaluated through unscheduled DNA synthesis (UDS) in A549 lung epithelial cells exposed to 10 μg/cm² of PM_{10} and 1 μM of BaP. Amplified panels of representative images of NER pathway activity after 48 h of exposure, recognized in cell that incorporated thymidine analogue (EdU) forming repair foci. Non-specific incorporation during DNA synthesis was identified by green homogeneous nucleus staining and these cells were not considered in the cell count for the repair analysis.
